# The effect of vibrotactile feedback on performance, perception and trust when balancing in different analog g-levels

**DOI:** 10.1007/s00221-025-07098-5

**Published:** 2025-05-31

**Authors:** Vivekanand Pandey Vimal, Paul DiZio, James R. Lackner

**Affiliations:** 1 Ashton Graybiel Spatial Orientation Laboratory, Brandeis University, Waltham, MA USA; 2 Psychology Department, Brandeis University, Waltham, MA USA; 3 Volen Center for Complex Systems, Brandeis University, Waltham, MA USA

**Keywords:** Spaceflight analog, Martian-g, Lunar-g, 0-g, Sensory augmentation, Dynamic balance, Spatial disorientation

## Abstract

We studied orientation-dependent vibrotactile feedback (VF) as a countermeasure to spatial disorientation (SD) in spaceflight analog environments. In Experiment 1, participants balanced themselves in a dynamic motion simulator in Earth (1-g), Martian (0.38-g), Lunar (0.166-g) and 0-g analog conditions. One group (*n* = 13) had VF and the Control group (*n* = 13) did not. As g-level decreased, attitude control and subjective confusion worsened, for both groups. An exponential model fit both groups. The Control group became significantly worse than its 1-g baseline at 0.61 to 0.23-g. Based on these model fits, the VF group performed slightly better than Controls in 1-g, significantly better between 0.82 and 0.10-g, and their performance advantage increased towards 0-g. However, both groups reported similar levels of confusion in their sense of angular position and velocity across all g-levels. The VF group reported high trust in VF cueing as g-level decreased, despite their worsening performance and subjective confusion, highlighting a dissociation between the effectiveness of VF and cognitive trust in VF. Despite its benefits during hypo-g exposures, VF did not fully restore 1-g proficiency. In Experiment 2, we assessed whether a new group (*n* = 13) of participants given extended exposure with VF in the Lunar analog condition would achieve 1-g level performance. Initial performance and confusion deteriorated significantly relative to 1-g but then improved significantly until 1-g baselines were restored for most measures. However, signatures of SD, including attitude drift and positional confusion were still present. These results suggest that VF potentially would enhance dynamic vehicle control in spaceflight but may not fully eliminate SD.

## Introduction

On Earth, spatial disorientation (SD) is one of the leading causes of fatal aircraft accidents (Poisson and Miller 2014). Astronauts face physiological and psychological challenges in novel environments because our human sensory systems have not evolved to provide adequate vestibular orientation capabilities without training. SD can arise when there is inaccurate or attenuated perception and control of motion or attitude (Lackner 1992). During gravitational transitions, such as when landing on or departing the surface of the Moon or Mars, the otolith organs, which contribute to our sense of orientation, will go between detecting only inertial acceleration from self movements in 0-g to detecting the diminished gravitational orientation cues associated with the Moon (0.166-g) or Mars (0.38-g). Additionally, sustained spaceflight in 0-g will cause sensory reweighting of the otolith and other signals (Carriot et al. 2021; Clément et al. 2020). Both the variable gravitational background cues and the reweighting will significantly increase susceptibility to SD (Shelhamer 2015).

**Fig. 1:**
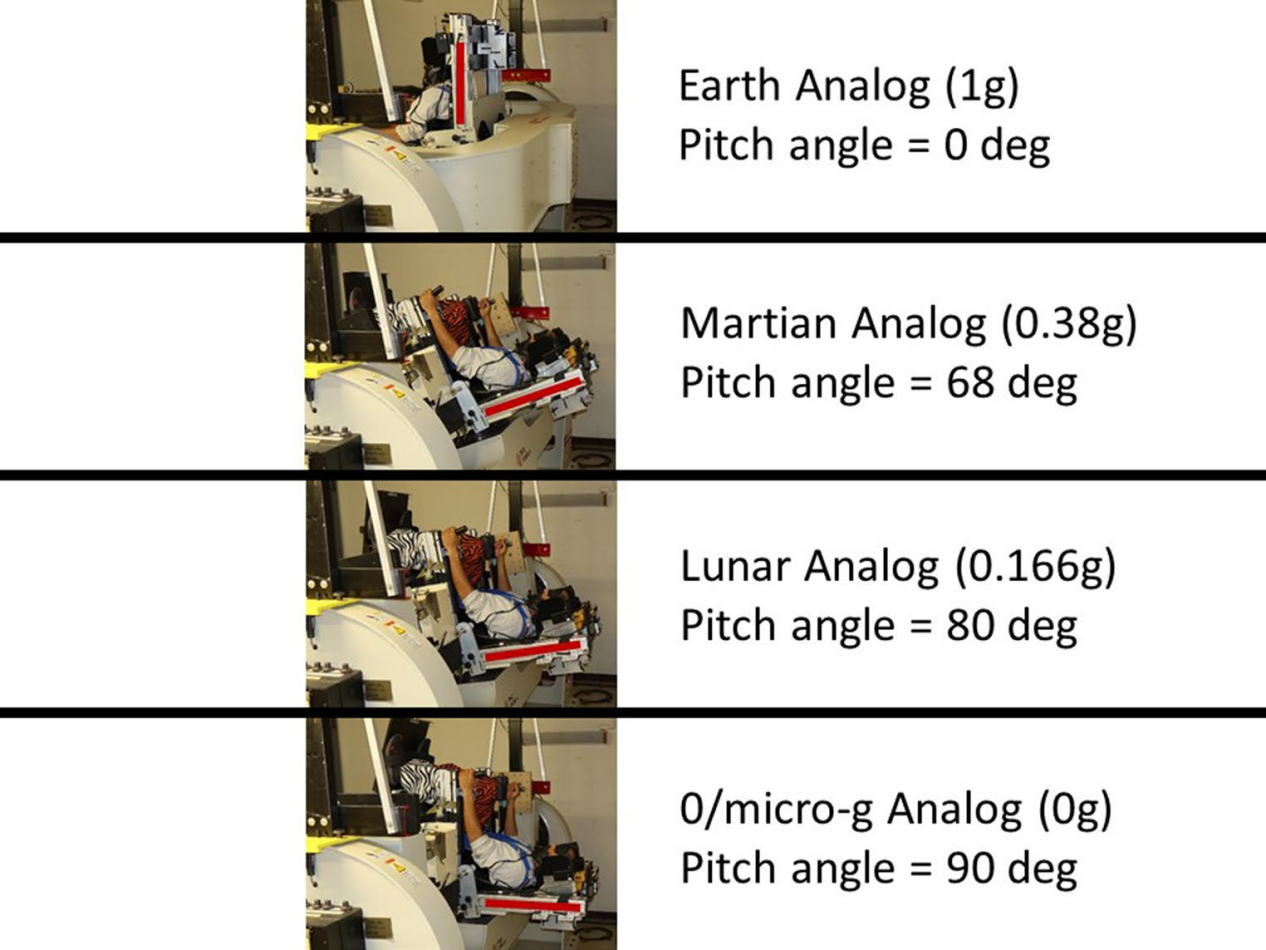
The multi-axis rotation device (MARS) was programmed with inverted pendulum dynamics at different pitched roll planes corresponding to the 4 different analog g-levels

Sensory augmentation, such as vibrotactile feedback (VF), is a potential countermeasure for SD. Information about body orientation is conveyed through small vibrating devices on the skin (Clément et al. 2018; Lawson and Rupert, 2014; Raj et al. 2000; Rupert 2000; van Erp et al. 2006). Recently, (Vimal et al. 2023), we evaluated VF effectiveness in a disorienting 0-g analog condition. Blindfolded participants riding in a Multi-Axis Rotation System (MARS) device programmed to behave like an inverted pendulum used a joystick to stabilize themselves about the balance point. In a 0-g analog condition, participants balanced in the supine roll plane where they did not tilt relative to the gravitational vertical and therefore could not use gravitational cues to determine their attitude relative to the balance point (Fig. 1). All 0-g analog (supine roll) participants experienced SD and showed poor performance and high rates of losing control relative to their performance in 1-g upright roll plane balancing. Participants in the 0-g analog (supine roll) condition who received orientation dependent VF performed significantly better than controls without cueing, but they showed limited learning and never reached the proficiency levels seen in the baseline Earth condition (upright roll). Many also reported confusion, with their perceived orientation being different from that indicated by the vibrotactors.

Based on these results, our first objective was to determine how blindfolded performance changes as g-level is decreased in the plane of balance performance. In Experiment 1, two groups (one with VF and one without) balanced in multiple pitched roll planes where the magnitudes of the gravitational cues in the balancing plane included Earth 1-g, Martian 0.38-g, Lunar 0.166-g and 0-g analogs (Fig. 1). All participants experienced all analog g conditions in a randomized order. Our second objective was to determine whether VF could restore performance back to Earth levels in these alternating partial-g conditions. Negen et al. (2023) have shown that VF can augment a noisy or diminished cue from another sensory system, suggesting to us that VF might potentially compensate for the diminished gravitational cues available in partial-g conditions.

Our third objective was to determine whether performance would improve with longer exposure to just the Lunar analog condition. Our prior work assessed transfer of 1-g training with a vibrotactile orientation feedback device to 0-g analog conditions (Vimal et al. 2023) because of the likelihood that training time would be limited in operational 0-g conditions. Participants with VF in the 0-g analog condition had shown learning across trials only when they had received prior specialized training in the Earth analog 1-g condition in which the balance point in different trials was randomly offset from the direction of gravity. This required them to rely on VF about the direction of balance. There was no improvement across trials in 0-g when prior 1-g training had not dissociated misleading natural gravitational cues from correct synthetic balance cues. In Experiment 2, we wanted to determine whether learning would occur in our *partial-g*, Lunar analog condition with VF, without the need for prior 1-g specialized training. Specifically, would extended exposure time with VF in the Lunar condition restore balance performance to baseline 1-g proficiency?

## Materials and methods

### Participants

39 healthy participants were recruited, with 13 in the Control (no VF) group (5 female and 8 male, 24 ± 9 years old) and 13 in the Experimental (VF) group (5 female, 8 male, 22 ± 4 years) of Experiment 1, and 13 in the Lunar Learning with VF group (5 female, 7 male, and 1 nonbinary, 21 ± 3 years) of Experiment 2. None had prior experience in the MARS or with VF. All signed an informed consent form approved by the Brandeis Institutional Review Board.

### Apparatus

The MARS device was programmed with inverted pendulum dynamics, $\:\ddot{\theta}={\text{k}}_{\text{P}}\text{sin}{\uptheta\:}$, where θ is the angular deviation from the direction of balance in degrees, and k_P_ is the pendulum constant. As in our prior work, we chose a challenging pendulum constant, 600 deg/s^2^_,_ ≈ 0.52 Hz (Vimal et al. 2017, 2018, 2019, 2023). A velocity increment proportional to joystick deflection was added to the MARS velocity at every time step (~ 50 Hz) and then integrated by a Runge-Kutta RK4 solver to calculate the new MARS angular position and velocity. Crash boundaries were set at ± 60 deg from the direction of balance, at which the MARS would stop and automatically reset to the start point. Angular velocity was limited to ± 300 deg/s and angular acceleration to ± 180 deg/s^2^. More information on the control scheme can is available in Panic et al. (2015). Ambient sound was masked by ear plugs and noise-cancelling earphones that played white noise, periodically interrupted by voice commands as described below.

### Analog g conditions

In the Earth analog condition, participants balanced in the upright roll plane where the balance point was aligned with the gravitational vertical. We refer to this as the 1-g condition, and we specify partial-g analog conditions as a proportion of 1-g. To diminish the strength of the gravitational cue in the roll plane, we pitched participants backwards. The pitch angle that corresponded to a g-analog was $\:{\text{cos}}^{-1}\left({g}_{analog}\right)$. For the Mars analog condition (g_analog_=0.38-g) participants were pitched back by 68.67 deg, for the Lunar analog condition (g_analog_=0.166-g) pitched back by 80.45 deg, and for the 0-g analog condition pitched back by 90 deg (Fig. 1).

### Vibrotactile feedback

The Experimental group in Experiment 1 and the Lunar Learning group in Experiment 2 received VF from 4 vibrotactors (Engineering Acoustics model C-2) attached by elastic bandages to each arm at equal intervals from the shoulder to the wrist. The vibrotactors were programmed to turn on, one at a time, on the side of MARS deviation from the balance point. The first vibrotactor (at the shoulder) activated when the MARS deviated beyond 1 deg from the direction of balance, the second beyond 7 deg (between the shoulder and elbow), the third beyond 15 deg (between the elbow and wrist), and the fourth, at the wrist, beyond 31 deg.

### Procedure

#### Experiment 1

Participants were given an overview of the experiment and shown videos of a person balancing the MARS in the Earth, Martian, Lunar and 0-g conditions. They were informed that the MARS behaved like an inverted pendulum and had crash boundaries at ± 60 deg from the direction of balance, after which the MARS would automatically return to the start point. They were instructed, “to avoid crashing, to stay near the balance point, and to minimize oscillations.” Those who were to receive VF were told how the vibrotactors worked. Once they had signed the consent form, they were secured in the MARS using a lap belt, a five-point safety harness, side torso panels, and a U-shaped, foam-lined head restraint with an embedded noise-cancelling headset. Participants were then told how the Logitech Freedom 2.4 cordless joystick attached to the right armrest controlled the MARS motion, and how the “kill switch” on the left armrest could be pressed to immediately stop the experiment. Before data collection began, they were blindfolded, the room was darkened.

Trials began with an auditory “begin” command that participants heard through the headset. During the trial, whenever the MARS reached the crash boundary ± 60 deg from the balance point, the participant heard “lost control, resetting” and the joystick was disabled until the MARS had automatically reset at a rate of 5 deg/s to the balance point, at which point the participants heard a “begin” command. Trials lasted 100 s of total balance time which excluded the reset times after crashes. However, if the total trial length reached 120 s, it was stopped.

The Control (no VF) and Experimental (with VF) groups underwent 20 trials divided into 5 blocks of 4. Blocks 1 and 2 were in the Earth analog condition so that participants could become familiar with controlling the MARS. Our prior work showed that 2 blocks are usually sufficient for learning after which performance begins to plateau (Vimal et al. 2016, 2019, 2020). Blocks 3–5 all contained 1 trial each of Earth, Martian, Lunar and 0-g analog conditions, in randomized order. Between blocks, participants were given a 2-minute break in the upright orientation, during which they were asked questions about their perceptual experiences, described below.

#### Experiment 2

Participants were given the same instructions as in Experiment 1 (avoid crashing, stay near the balance point, and minimize oscillations), but were only shown training videos of participants balancing in the Earth and Lunar conditions. All participants received VF in 24 trials divided into 6 blocks of 4. Blocks 1 and 2 were in the Earth analog condition. Blocks 3–6 were in the Lunar analog condition. Participants were given a 2-minute break between blocks in the upright orientation during which they were asked questions about their perceptual experiences, as described below.

### Subjective ratings

In Experiment 1, at the end of Blocks 3–5, participants were asked for ratings of each of the 4 preceding trials, separately for Earth, Lunar, Martian, and 0-g. Both groups rated their positional confusion (PosConf) and velocity confusion (VelConf) where 1 was ‘not confused’ and 10 was ‘very confused, I have no idea where I was’. The Experimental Group, who had VF, also rated their Trust, from 0 to 100, that the vibrotactors were correctly indicating the balance point, and rated whether the VF was Useful, where − 10 meant that it was distracting, 0 meant it did not help or hurt, and 10 meant that it was very useful and they wouldn’t be able to balance without it. Participants in Experiment 2, after Blocks 3–6 were asked to make the same ratings about the preceding 4 Lunar-g trials as a unit.

### Data analysis

MARS angular position and velocity data were sampled at 20.7 ± 1.1 msec (approximately 50 Hz) and passed through a zero-phase 5-pole high-pass Butterworth filter with a cutoff frequency of 5 Hz. After filtering, data from the reset periods following crashes were removed. For each trial, we calculated four performance metrics for the MARS position and velocity, and two joystick command metrics from the joystick deflection records.

### MARS performance

Crash frequency (Crashes) was defined as the number of crashes in a trial divided by the duration of the trial multiplied by 60 to obtain units of min^-1^. To measure the ability to stay near the upright, we computed the mean of the absolute value of MARS deviation from the direction of balance (0 deg),|Mag|_Pos_. We also computed the rate of positional drift (DriftRate) by tracking successive MARS oscillations between crashes (cf. Vimal et al. 2020 for more details). Such drift is a characteristic feature of SD in the 0-g analog condition (Vimal et al. 2016, 2017, 2018, 2019). To quantify the ability to minimize positional variability, we calculated the standard deviation of MARS angular position (STD_MARS_).

### Joystick commands

We calculated the average of the absolute value of joystick deflection angle (|Mag|_Joy_) across each trial,. Joystick inactivity (%Zero) was calculated as the percentage of data points where joystick deflection was ≤ ± 1% of its maximum amplitude. More intermittent, smaller magnitude joystick movements are seen in MARS conditions which are less challenging or where participants have become more proficient (Vimal et al. 2018; Vimal et al. 2019; Vimal et al. 2023).

## Results

### Experiment 1

**Fig. 2:**
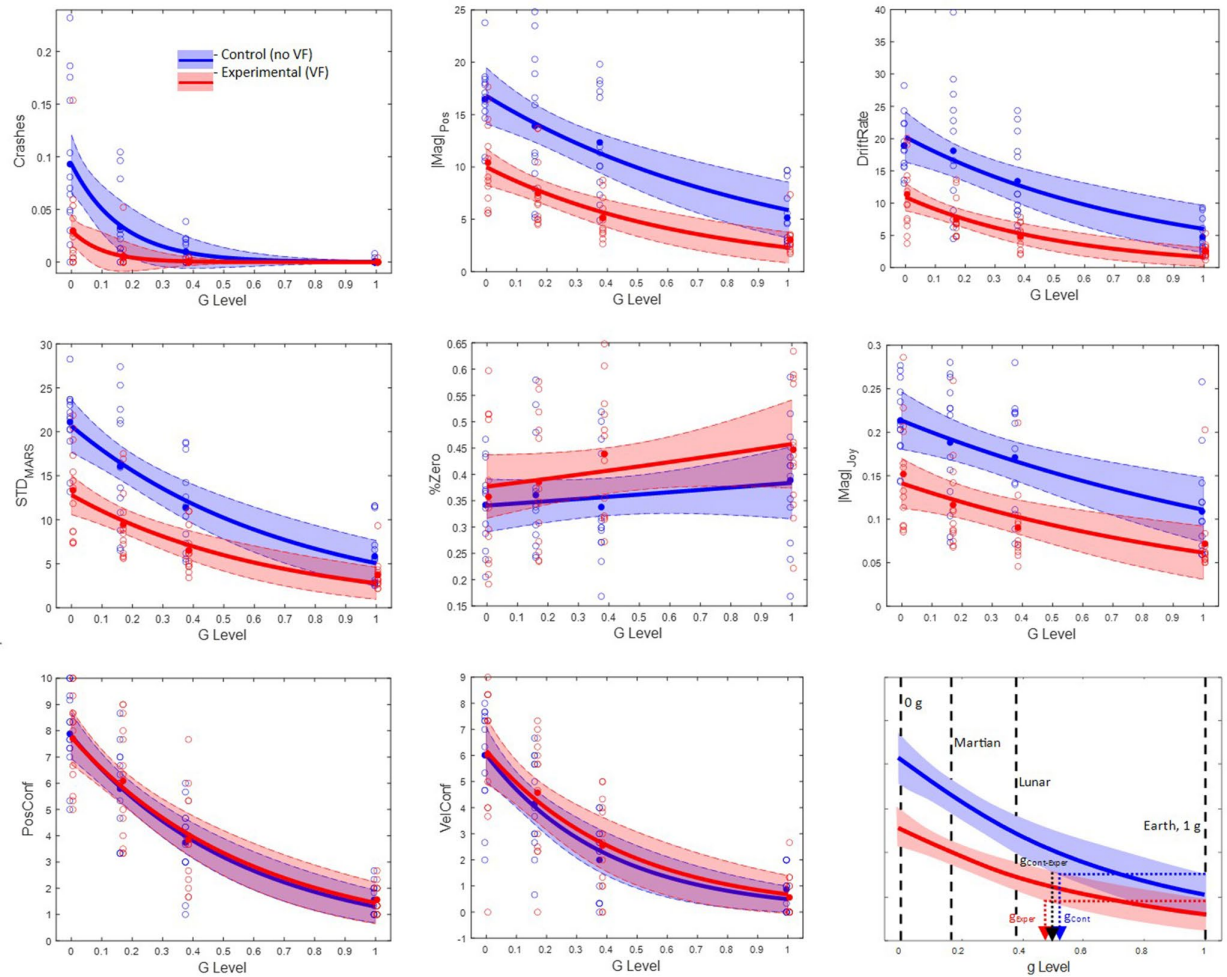
Exponential fits applied to measures of MARS performance (Crashes,|MARS|_Pos_, DriftRate, STD_MARS_), joystick commands (%Zero,|Mag|_Joy_), and subjective confusion (PosConf, VelConf) as a function of analog g-level, in Experiment 1. In blue is the Control group and in red is the Experimental group. Open dots are all individual participants, solid lines are the fitted values, and colored bands are prediction intervales. The lower right panel illustrates how the prediction intervals were used to evaluate the divergence of the Control and Experimental group (g_Cont−Exper_) as well as the evolution of performance degradation in each group due to reduced g level (g_Cont_, g_Exper_), for a hypothetical, prototypical variable

We examined in Experiment 1 how different simulated g-levels and the presence or absence of VF affected MARS performance, joystick command style, and subjective ratings. Prior to statistical analysis, all dependent variables described above were averaged across Blocks 3–5, per analog-g level. Blocks 1–2 were the 1-g training condition and were not analyzed. The top left panel of Fig. 2 illustrates a trend of increasing Crashes as g-level decreases; the other three MARS variables (|Mag|_Pos_, DriftRate, STD_MARS_) show similar performance degradation as the magnitude of the analog gravitational acceleration decreased. These degradation trends were evident for every individual participant in both the Experimental and Control groups. The prevalence of joystick inactivity (%Zero) decreased as g-level decreased and the magnitude of joystick activity (|Mag|_Joy_) increased in a complementary trend. The two subjective estimates of confusion (PosConf and VelConf) increased as g-level decreased. Trust and usefulness (obtained only for the Experimental VF group) appeared constant across the 4 analog g-levels.

We initially compared the VF and Control groups with independent t-tests at each analog g-level. Table 1 shows that VF significantly improved performance when compared to the Control group especially in partial-g analog conditions. To obtain a better resolution of the advantage conferred by vibrotactile cues, we performed exploratory rectilinear (first order polynomial) and exponential (single exponential with zero asymptote) fits as a function of analog g-level (4 g-levels x 13 participants per group), for the 8 variables obtained in both groups, using the MATLAB *fit* function. For commensurability, both models were processed with a non-linear least squares criterion. The variance accounted for (r^2^ value) was higher for the exponential than the linear fit for every dependent measure for both participant groups. Therefore, subsequent analyses focused on the exponential model. Fifteen of the 16 exponential fits (8 variables x 2 groups) were significant; Only %Zero in the Control group was not significant (the fit was no better than the mean across g-levels). See Table 2. As g-level decreased, both groups showed significant declines in performance, increases in confusion, and changes in joystick activity.

**Table 1:** Results of independent t tests comparing the vibrotactile feedback versus control for each analog g-level in experiment 1

Metric	0-g	Lunar-g	Mars-g	Earth, 1-g
Performance				
Crashes	**0.012**	0.018	0.015	0.41
|Mag|_Pos_	**0.00050**	**0.0042**	**0.00010**	0.023
DriftRate	**0.00080**	**0.0018**	**0.0001**	**0.011**
STD_MARS_	**0.0030**	**0.0071**	**0.0038**	0.070
Joystick Command				
%Zero	0.73	0.58	0.03	0.22
|Mag|_Joy_	**0.0079**	**0.0091**	**0.0010**	0.074
Confusion Rating				
PosConf	0.79	0.71	0.88	0.96
VelConf	0.97	0.61	0.40	0.37

The p-levels have been adjusted (Bonferroni) for 4 comparisons per analog g-level. Bold font highlights significant adjusted results, *p* <.05

**Table 2:** Statistics of the exponential model fit to changing performance, joystick, and confusion metrics as function of g-level - $\:\widehat{\text{y}}\:$= a * exp(b*x), where X is 0 g to 1-g, in experiment 1

Metric	*r* ^2^_Cont_	*r* ^2^_Exper_	g_Cont_	g_Exper_	g_Cont-Exper_
Performance					
Crashes	**0.455**	**0.238**	0.23	0.07	0.1
|Mag|_Pos_	**0.453**	**0.475**	0.45	0.46	0.82
DriftRate	**0.416**	**0.486**	0.40	0.44	0.8
STD_MARS_	**0.570**	**0.490**	0.51	0.46	0.50
Joystick Command					
%Zero	0.026	**0.062**	- ^1^	-	-
|Mag|_Joy_	**0.298**	**0.243**	0.35	0.30	0.69
Confusion Rating					
PosConf	**0.747**	**0.661**	0.61	0.55	-
VelConf	**0.615**	**0.534**	0.49	0.43	-

The r^2^ values are bolded if the exponential fit is significant (better than using the mean of the predicted metric). The last three columns represent, respectively, g values at which the confidence interval for the Control group falls outside its baseline value (g_Cont_), the Experimental group falls outside its baseline 1 g range (g_Exper_), and Experimental group falls outside the Control range (g_Cont−Exper_)

^1^ “-“ indicates confidence intervals did not diverge

To compare the Control and Experimental conditions, we started by computing the 95% prediction intervals for the exponential fits (MATLAB *predint* function), per variable, per condition, which are plotted in Fig. 2. To determine, for the Control group, the g-level at which performance deteriorated, joystick activity changed, and confusion increased significantly, we computed the g-level at which the prediction interval for each variable fell entirely outside its baseline 1 g prediction interval. This value is illustrated for a hypothetical variable in the lower right panel of Fig. 2, with the arrow labelled g_Cont_. The values of g_Cont_ for all variables are listed in Table 2. To determine where the changes became significant in the Experimental group, we computed the g-level at which the prediction intervals fell entirely outside their baseline 1-g prediction intervals. See g_Exper_ in Fig. 2; Table 2. To determine the analog g-level at which VF began to ameliorate performance degradation, to alter joystick commands, and to decrease confusion relative to Control performance, we computed the g-level at which the prediction intervals of the Experimental and Control groups first became non-overlapping. See g_Cont-Exper_ in Fig. 2; Table 2.

The plots in Fig. 2 show that both groups’ MARS performance deteriorated approaching the 0-g space flight analog condition and the Experimental group performed better than the Control. The g_Cont-Exper_ statistics in Table 2 indicate that the Experimental group became significantly better than Controls at g-levels ranging from 0.82-g for|Mag|_Pos_, 0.80-g for DriftRate, 0.5-g for STD_MARS_, and 0.1-g for Crashes. At g-levels below these divergence points, the magnitude of the significant advantage for the Experimental group increased. The g_Cont_ and g_Exper_ statistics show that although both groups’ performance overlapped at 1-g and deteriorated as g-level decreased, the Experimental group fell below its 1-g baseline at lower g-levels than the Control group. The advantage of vibrotactile cueing became significant between 0.61-g to 0.23-g and grew as g-level was reduced. These results hold for measures reflecting all three MARS performance instructions - “to avoid crashing” (Crashes), “to stay near the balance point” (|Mag|_Pos_, DriftRate), and “to minimize oscillations” (STD_MARS_).

The joystick commands followed similar trends to MARS performance. In both groups, the rate of inactivity (%Zero) decreased at lower analog g-levels, and, accordingly, the net magnitude of joystick commands (|Mag|_Joy_) increased. Thus, the more challenging space flight analog conditions resulted in larger, more frequent joystick actions. The less active, smaller magnitude joystick movements in the Experimental group are consistent with vibrotactile cueing reducing the challenge at each g-level. Only|Mag|_Joy_ showed significant divergence between groups, with vibrotactile cueing becoming lower at and below 0.69-g.

Both confusion ratings increased significantly with decreasing g-level but neither showed benefit from vibrotactile cueing, in contrast to the VF benefits for performance and joystick measures. The measure of Trust in the VF cueing hovered between 80 and 85% and Usefulness between 8 and 10, but neither showed a significant effect of g-level, in contrast to the deterioration of MARS performance and the increasing confusion.

### Experiment 2

**Fig. 3:**
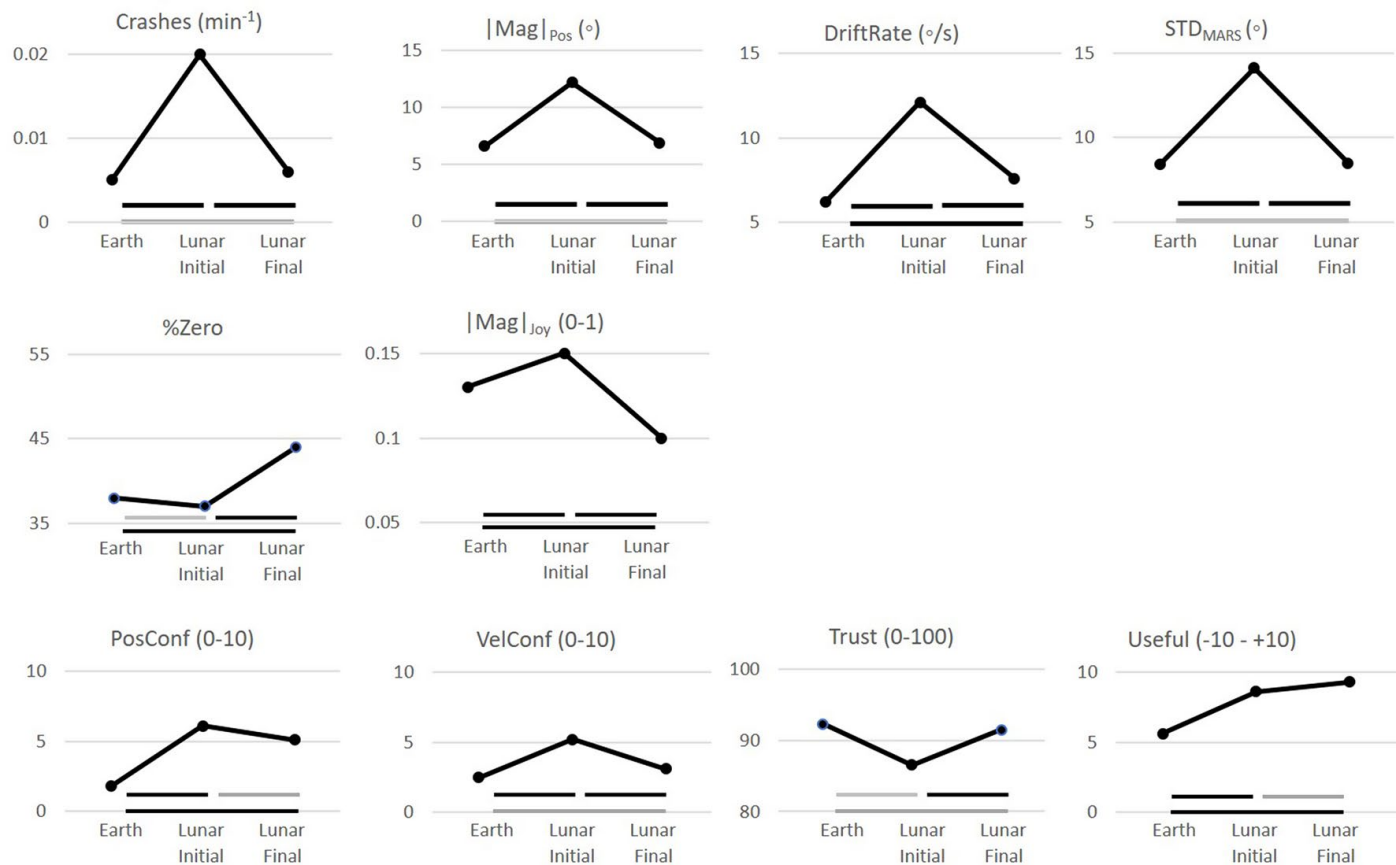
Comparison of vibrotactile cueing effects in Experiment 2, across blocks of 1 g Earth conditions (Earth), acute exposure to analog Lunar gravity (Lunar Initial), and after prolonged Lunar g (Lunar Final). The horizontal bars spanning conditions encode whether paired t tests are significant (black, *p* <.05 at least) or not (gray)

In Experiment 2, we examined whether greater exposure time in the Lunar condition with VF would lead to further improvements in performance and perception. Figure 3 plots the mean of every variable during vibrotactile cueing in the second block of 4 trials (1-g, Earth), the third block (Lunar-Initial: initial exposure to Lunar-g analog), and sixth block (Lunar-Final: final exposure to Lunar-g). Inspection of Figs. 2 and 3 reveals that the values for all dependent variables in the 1-g Earth baseline and the initial block of the Lunar analog of Experiment 2 closely replicated those in the comparable conditions for the Experimental group of Experiment 1. The four MARS performance variables showed initial degradation at the beginning of Lunar-g exposure (Lunar Initial) and recovery toward the 1-g baseline by the end of Lunar-g exposure (Lunar Final). The horizontal bars below each plot in Fig. 3 encode whether paired t-tests between the spanned conditions are significant. The t-tests showed that Crashes, positional deviation from the upright (|Mag|_Pos_), positional drift (Drift), and positional variability (STD_MARS_) all increased significantly between 1-g (Earth) and initial Lunar-g exposure (Lunar Initial) and then decreased significantly at the end of Lunar-g exposure (Lunar Final). Only DriftRate remained slightly but significantly elevated relative to the 1-g baseline after the final Lunar block (Lunar Final). The variables reflecting the joystick commands underlying MARS performance changed slightly but significantly upon transition from Earth 1-g to Lunar-g and showed significant recovery over the period of Lunar-g exposure. The t tests showed significantly greater inactivity (%Zero) and smaller amplitude (|Mag|_Joy_) in the final Lunar-g exposure (Lunar Final) relative to 1-g baseline (Earth). In Experiment 1, more challenging acute Lunar analog conditions had resulted in larger and more frequent joystick actions; the significantly smaller and more infrequent movements after prolonged Lunar exposure in Experiment 2 are consistent with vibrotactile training reducing the challenge of operating the MARS in Lunar-g.

Positional and velocity confusion increased initially, simultaneously with a reduction in trust of the vibrotactors (Trust) and a paradoxical increase in subjective assessment of their utility (Useful). All except the reduction in trust were significant. Positional confusion did not decrease with prolonged Lunar-g vibrotactile cueing, but velocity confusion and trust in the vibrotactors fully recovered to their baseline values, while subjective usefulness remained significantly elevated.

## Discussion

### The effect of different g-levels on performance and perception

In Experiment 1, participants balanced in Earth, Martian, Lunar and 0-g analog conditions. The Experimental group had VF while the Control group did not. For both groups, as g-level decreased, performance significantly worsened as indicated by increases in positional error, positional variability, the frequency of crashes, the frequency and magnitude of joystick deflections, and the rate of positional drift which our prior work has shown is a marker of spatial disorientation (SD) (Vimal et al. 2017, 2018, 2019, 2020). As g-levels decreased, both groups’ ratings of positional and velocity confusion increased (Fig. 2). Even at a partial gravity level with appreciable gravitational cues - Martian-g - performance became significantly worse and SD increased, suggesting that even small a decrease in the magnitude of gravitational cues will have a significant effect on performance and perception. Work in other fields has shown that even slightly diminished or noisy sensory cues can quickly lead to deficits in performance (Wingfield et al. 2005).

### The effect of vibrotactile feedback on performance and perception at different g-levels

In Experiment 1, as g-level decreased, the magnitude of improvement from the VF increased. The Experimental group performed significantly better than the Control group for g-levels even greater than Martian-g (0.38 g), showing less positional variability and positional drift, and better joystick control (Table 2). By 0-g, the Experimental group also had significantly fewer crashes. All of these performance improvements were acquired after limited experience with VF - only 8 100-second trials of exposure in the Earth condition in Blocks 1 and 2. This suggests that VF could be an effective countermeasure for SD in a dynamic orienting task at partial and 0-g analog conditions. It is important to recognize that the efficacy of tactile cueing, or lack of it, varies depending on the nature of the tactile-spatial coding, the orientation task, and the context (Harris et al. 2017; Lawson et al. 2016; Peterka et al. 2006; Reuten et al. 2023; van Erp and van Veen 2006; van Erp et al. 2007).

Vimal et al. (2023) had found that participants reported high levels of both positional and velocity confusion in the 0-g analog condition compared to the Earth analog condition. This was true for groups both with and without VF. We had wondered whether in partial-g conditions the VF would augment the diminished gravitational cues sufficiently that participants would not experience any positional and velocity confusion. However, we found no difference between the Control and Experimental groups: VF did not enhance perception or reduce the magnitudes of subjective positional and velocity confusions. One explanation for positional confusion could be that neurons that respond to both otolith and semicircular canals signals (Hupfeld et al. 2022) received less input from the otoliths and led to the perception of orientation and movement being less. By contrast, no vestibular model predicts the velocity confusion that every participant reported. Our findings suggest that initial exposure to partial or 0-g environments, such as during g-transitions, will not only impact perception of positional orientation but also perception of velocity which is an understudied contributor to SD.

### The effect of extended exposure on performance in the lunar analog

In Experiment 1, VF did not restore performance back to Earth levels, perhaps because participants only had 3 intermixed trials of each analog condition and therefore had little opportunity to learn. Our goal in Experiment 2 was to see whether ​extended exposure to VF during diminished gravitational cues in a Lunar analog condition would eliminate performance degradation. In Experiment 2, participants had VF and first experienced 8 trials in the Earth analog condition to become familiarized with the vibrotactors and then had 16 consecutive trials in the Lunar analog condition. We found that as participants transitioned from the Earth condition (Block 2) to the Lunar condition (Lunar Initial, Block 3), performance significantly worsened, confirming the findings in Experiment 1 (Fig. 2). From Block 3 to 6 in the Lunar analog condition, participants showed significant learning. They were able to reduce their positional error, positional variability and positional drifting which is a marker for SD. They also displayed better joystick control by decreasing joystick magnitudes and increasing their intermittency of joystick commands. Our prior work shows these are signs of proficiency. This led to a reduced frequency of crashes. By Block 6 in the Lunar analog condition, participants were performing at the level of the Earth analog (Block 2) and even better for some metrics. Only the magnitude of positional drift and ratings of positional confusion were worse. These results show that extended exposure in a partial-g environment with VF augmentation is sufficient for significant improvements in performance, though measures of SD remain unimproved such as positional drifting and positional confusion.

### Why are participants with vibrotactile feedback able to show significant learning in the lunar analog but not in the 0-g analog without specialized training?

In the 0-g analog, the missing positional gravitational information normally sensed by the otolith organs has to be replaced by the VF. The sensory substitution literature suggests that this is possible when participants are first given time to freely explore with the device (active sensing). However, in Vimal et al. (2023) even after providing an entire experimental session for active sensing in the Earth analog, participants failed to show continued learning when then placed in the 0-g analog condition with VF. This discrepancy from the sensory substitution literature is likely because most prior studies had trained and tested participants in the same environment, whereas in Vimal et al. (2023), participants trained in one condition (Earth analog) and then were tested in a novel one (0-g analog). We found that in addition to active sensing, participants also needed to learn how to disengage from their vestibular sense of position while focusing on their VF. After providing this specialized training, participants showed continued learning.

In contrast to the 0-g condition where there were no task relevant gravitational cues, in our partial-g analog conditions there were diminished positional gravitational cues detected by the otolith organs, which were augmented by the VF. In these conditions, we found that active sensing without prior specialized training allowed learning in the Lunar analog condition (Fig. 3). A possible explanation is that in the presence of the diminished gravitational cues the VF assisted in reweighting the otolith signal, possibly amplifying its contribution.

### The effect of extended exposure on perception in the lunar analog

In Vimal et al. (2023) and in the Lunar analog condition of Experiment 1, we found that participants consistently reported high levels of position and velocity confusions. Prolonged exposure to the Lunar condition changed this. By the end of Experiment 2, participants reported a significant decrease in their velocity confusion ratings that reached the Earth analog level. These findings are important because they reveal that initial exposure to partial or 0-g environments may lead to misperception of velocity cues and therefore could be an unexpected contributor to SD. However, our findings indicate that, at least in partial-g environments, with enough exposure time, perception of velocity can come back to Earth levels. The change in the perception of velocity confusion may be further evidence that some form of sensory reweighting is occurring where the semicircular canal signal may be amplified across trials or potential somatosensory cues may be recruited.

In Vimal et al. (2023), even with greater exposure time in the 0-g analog condition, VF did not reduce the high ratings of positional confusion. Would VF during extended exposure in the Lunar condition where there are diminished gravitational cues lead to a change in confusion? In Experiment 2, we found that the positional confusion did not change even as performance improved across trials. Therefore, while there were performance improvements in MARS position control, the lack of improvements in positional confusion suggests that full sensory integration is not occurring, but perhaps some form of sensory addition where the VF may be interpreted as a new sense (Sienko et al. 2018).

### Trust vs. reliance

In Experiment 1, participants reported high levels of trust (80–85%) in VF which did not change across different g-levels. These high levels of trust in the vibrotactors did not translate to restored performance to Earth levels when exposed to partial-g and to 0-g analog conditions, which is consistent with the findings in Vimal et al. (2023). “Cognitive trust” may be different from “sub-cognitive trust” in a sensory augmentation device, especially in challenging tasks. Other studies have shown that sub-cognitive connection with a sensory augmentation device during novel conditions requires some amount of sensory reweighting (Bao et al. 2018; Wildenberg et al. 2010, 2011). Our results suggest that to build sub-cognitive trust in partial-g conditions requires extended exposure in partial-g environments with the cueing system (Experiment 2). However, in the absence of gravitational cues, in 0-g, our prior work shows that to build sub-cognitive trust requires not only active sensing but also training participants how to disengage from one sensory system while focusing on the sensory feedback from the cueing device (Vimal et al. 2023).

### Limitations and future directions

While our analog conditions are able to capture some aspects of spaceflight related SD, one limitation is that we are unable to reproduce the sensory reweighting that will occur in the vestibular system as a result of long duration spaceflight. These changes will likely cause even greater SD than is seen in our Control group findings. Spaceflight will also introduce many tasks that astronauts and pilots will have to simultaneously perform. In the future, we will conduct dual task experiments in our disorienting analog conditions to determine whether VF enhances performance because it operates through a different sensory modality (Sklar and Sarter 1999), or whether it adds too much cognitive load and reduces performance.

Another limitation is related to our findings that velocity confusion decreased with greater exposure to the Lunar-g analog. An alternative possibility may be that as performance improved with greater exposure it influenced participants’ perception of their velocity confusion. In the future, we will pair extended exposure in the Lunar-g condition with a dedicated perceptual learning paradigm (Fitze et al. 2024) to determine whether perception of velocity will improve with greater exposure time.

On the topic of training, we are interested in determining how to reduce the positional confusion resulting from VF. In future experiments, we will explore whether long exposure to the Lunar-g analog condition will result in a reduction in positional confusion or whether specialized training such as in Vimal et al. (2023) is essential. We will also examine whether more naturalistic cues that influence the perception of orientation, such as pressure cues felt on the sides of the body, can correct the perceptual errors caused by disorientation. Often pilots are unaware that they are disoriented until it is too late (Stott 2013). The unique feature of VF is that it provides continuous information about attitude without needing visual attention. In future experiments we will determine whether a trained pilot can use the discrepancy between their erroneous perception of orientation and their correct orientation indicated by VF as an early detection of their SD.

## Data Availability

No datasets were generated or analysed during the current study.
